# Ultrastructural alterations in *Schistosoma mansoni* juvenile and adult male worms after in vitro incubation with primaquine

**DOI:** 10.1590/0074-02760160324

**Published:** 2017-03-02

**Authors:** Reem Osama A Kamel, Fatma El-Zahraa Anwar Bayaumy

**Affiliations:** Ain Shams University, Women College for Arts, Science and Education, Department of Zoology, Asmaa Fahmey St., Cairo, Egypt

**Keywords:** Schistosoma mansoni, primaquine, Praziquantel, schistosomicidal activity, tegumental alteration

## Abstract

**BACKGROUND:**

Praziquantel has been cited as the only drug for treating schistosomiasis. However, concerns over drug resistance have encouraged the search for novel drug leads. The antimalarial drug primaquine possesses interesting anti-schistosmal properties.

**OBJECTIVES:**

This study is the first to document the potential role of primaquine as a schistosomicide and the ultrastructural changes induced by primaquine on juvenile or adult male worms of *Schistosoma mansoni*.

**METHODS:**

Ultrastructural alterations in the tegumental surface of 21-day-old juvenile and adult male worms of *S. mansoni* were demonstrated following primaquine treatment at different concentrations (2, 5, 10, 15, and 20 µg/mL) and incubation periods (1, 3, 6, 24, and 48 h) in vitro, using both scanning and transmission electron microscopy.

**FINDINGS:**

At low concentrations (2, 5, and 10 µg/mL) both juvenile and adult male worms were alive after 24 h of incubation, whereas contraction, paralysis, and death of all worms were observed after 24 h of drug exposure at 20 µg/mL. The tegument of juvenile and adult male worms treated with primaquine exhibited erosion, peeling, and sloughing. Furthermore, extensive damage of both tegumental and subtegumental layers included embedded spines, and shrinkage of muscles with vacuoles. The in vitro results confirmed that primaquine has dose-dependent effects with 20 µg/mL as the most effective concentration in a short incubation period.

**MAIN CONCLUSIONS:**

The schistosomicidal activity of primaquine indicates that this drug possesses moderate in vitro activity against juvenile and adult male worms, since it caused high mortality and tegumental alterations. This study confirmed that the antimalarial drug primaquine possesses anti-schistosomal activity. Further investigation is needed to elucidate its mechanism of action.

Schistosomiasis is a chronic disease with high prevalence and wide distribution worldwide. It is endemic in 76 countries distributed throughout Africa, Southeast Asia, and Central and South America (Mata-Santos et al. 2014). It affects more than 200 million people worldwide, and nearly 800 million others are at risk of infection (Colley et al. 2014).

Due to unavailability of a schistosomiasis vaccine, the common strategy for treatment and control is mainly based on chemotherapy (Pereira et al. 2015). Praziquantel (PZQ) is the drug of choice for large-scale treatment of schistosomiasis as it is a safe and cheap therapy (Cheng et al. 2011). The drug targets the adult worm, but has minor activity against the young developing stages (i.e., schistosomula); hence, repeated treatment is necessary to kill the parasites that have matured (Doenhoff & Pica-Mattoccia 2006). Unfortunately, drug resistance is still a threat in long-term administration of PZQ. Therefore, new chemotherapies against schistosomiasis are desperately required (Doenhoff et al. 2008).

Both anti-malarials and their derivatives may have a therapeutic impact in early and chronic schistosomiasis (Mitsui & Aoki 2010). Interestingly, the antimalarial drug, mefloquine, is reported to be effective against both *Schistosoma mansoni* and *Schistosoma japonicum* in vivo and in vitro (Manneck et al. 2010, Mossallam et al. 2015). Furthermore, it was demonstrated that not only mefloquine, but also other antimalarial drugs such as amodiaquine, primaquine and chloroquine exhibited in vitro anti-schistosomal activity. In addition, schistosomiasis and malaria share roughly the same epidemic regions (Mitsui & Aoki 2010). Thus, it has been confirmed that the anti-malarial drug primaquine displays anti-schistosomal properties against both juvenile and adult worms of *S. mansoni* in vitro, causing a pronounced deformation of the parasite body (Holtfreter et al. 2011). Furthermore, primaquine was found to affect the lysosomal acidic vesicles of the schistosomula involved in endocytosis and detoxification (Carneiro-Santos et al. 2001). Moreover, Mitsui and Aoki (2010) reported that primaquine inhibited the daily egg output and decreased the survival of both male and female worms.

The tegument of an adult schistosome is a protective sheath that plays a role in defence as well as in the uptake of nutrients, osmoregulation, and excretion. Although alterations in the surface ultrastructure of schistosome worms have received little attention, several investigators have used them for evaluating anti-schistosomal drugs (El-Shabasy et al. 2015). As male worms are more sensitive to anti-schistosomal drugs than females are (Shaw & Erasmus 1987), the current study is the first to document the alterations in the tegumental surface of 21-day-old juvenile and adult male worms of *S. mansoni* following primaquine treatment at different concentrations in vitro by a detailed temporal examination using both scanning and transmission electron microscopy.

## MATERIALS AND METHODS

*Animals and parasites* - Male Syrian golden hamsters (*Mesocricetus auratus*) weighing 100-110 g were infected via shaved abdominal skin with 350 ± 10 cercariae of the Egyptian strain of *S. mansoni* per animal, freshly shed from experimentally infected *Biomphalaria alexandrina*. The animals and parasites were obtained from the Schistosome Biological Supply Centre (SBSC), Theodor Bilharz Research Institute (TBRI), Giza, Egypt. The hamsters were maintained on a standard commercial pellet diet and kept in an air-conditioned animal house at 20-22ºC. All experiments were approved by the animal ethics committee and carried out at the SBSC/TBRI, in accordance with the internationally valid animal ethics guidelines.

*Drugs* - Primaquine bisphosphate and Praziquantel were purchased from Sigma-Aldrich Chemical Co. (St. Louis, MO, USA). Thirty milligrams of primaquine was dissolved in 3 mL injectable water to prepare a stock solution of 10 mg/mL (Holtfreter et al. 2011). Further dilutions were carried out using aqua ad injectabilia. PZQ was dissolved in 0.1% dimethyl sulphoxide (DMSO) to be used as a reference drug.

*Culture medium and parasite preparation* - Juvenile worms were recovered from the hepatic veins of infected hamsters after 21 days post infection by perfusion (Clegg & Smithers 1972). Seven weeks following infection, adult *S. mansoni* worms were retrieved by perfusion of both the hepatic portal system and the mesenteric veins (Duvall & DsWitt 1967). All the recovered juvenile and adult male worms were washed several times from blood in small sieves (20 μM mesh) using phosphate buffer. They were then rinsed thrice with the culture medium used for the assay in a sterilised laminar flow chamber. The culture medium used was Roswell Park Memorial Institute medium (RPMI 1640) supplemented with 2 mM L-glutamine, 20% foetal calf serum, and antibiotics (300 μg streptomycin, 300 IU penicillin, and 160 μg gentamycin per mL).

*Assessment of the anti-schistosomal effect of primaquine on S. mansoni juvenile or adult male worms in vitro* - Juvenile or adult male worms of *S. mansoni* were subjected to treatment with varying concentrations of primaquine (2, 5, 10, 15, and 20 µg/mL). These concentrations were based on the plasma concentration levels during chemotherapy and prophylaxis (Elmes et al. 2006). The bioassay was carried out in 24-well culture plates (Costar) containing the same medium as previously mentioned (Xiao et al. 2014). Four juvenile or adult male worms were used in each well and two replicates were set up. Subsequently, the plates were incubated in a 5% CO_2_ incubator at 37ºC for 24 h (Xiao et al. 2007). The final volume in each well was 2 mL. Pure medium served as the negative control, whereas PZQ (5 µg/mL) was used as the positive control. Treated worms were examined for viability at intervals of 1, 3, 6, 24, and 48 h using an Olympus Inverted Microscope (Tokyo, Japan). The surviving parasites were identified as freely swimming without any structural deformation, whereas the dead parasites were recognised by complete loss of motility and were lying at the bottom of the well (Moraes 2012). The mortality rate of the worms was recorded for each concentration by calculating the number of dead worms relative to the total number of worms (Holtfreter et al. 2011, El Bardicy et al. 2012). The mean ± SD of three mortality values (of three independent experiments) for each concentration was also calculated at different times.

*Scanning electron microscopy of juvenile and adult male worms of S. mansoni incubated for 24 h with PZQ or primaquine* - Scanning electron microscopy (SEM) was used for the ultrastructural analysis of the effect of primaquine bisphosphate against *S. mansoni* juvenile or adult male worms. The juvenile or adult male worms incubated in pure medium (negative control), 5 µg/mL of PZQ (positive control), or those incubated with different concentrations (10, 15, and 20 µg/mL) of primaquine for 24 h, were washed several times with normal saline, fixed with 2.5% glutaraldehyde, and then dehydrated with serial dilutions of ethanol using an automatic tissue processor (Leica EM TP). Thereafter, the worms were dried using a CO_2_ critical point drier (Tousimis Audosamdri- 815). Specimens were coated with a gold sputter coater (SPI- Module). Coated worms were observed and photographed using a scanning electron microscope (JEOL- JSM - 5500 LV) under the high vacuum mode (Xavier et al. 2010) at the Regional Center of Mycology and Biotechnology, El Azhar University, Cairo, Egypt.

*Transmission electron microscopy of adult male worms of S. mansoni incubated for 24 h with PZQ or primaquine* - Primaquine bisphosphate-treated *S. mansoni* adult male worms at different concentrations (10, 15 and 20 µg/mL) and other adult male worms incubated in pure medium (negative control) or in 5 µg/mL PZQ (positive control) for 24 h, were fixed in 5% glutaraldehyde prepared in 0.1 M sodium cacodylate buffer for 4 h. Post fixation was performed with 1% osmium tetroxide in the same buffer for 2 h. Afterwards, the worms were dehydrated through a graded ethanol series before embedding in an epoxy resin. Ultrathin sections (50-80 nm thick) were double stained with uranyl acetate followed by lead citrate. The stained sections were examined and photographed with a JEOL 1010 Transmission Electron Microscope (Xavier et al. 2014) at the Regional Centre for Mycology and Biotechnology, Al-Azhar University, Cairo, Egypt.

*Statistical analysis* - The results of this study were calculated as mean ± SD using Microsoft Excel software.

## RESULTS

**Fig. 1:**
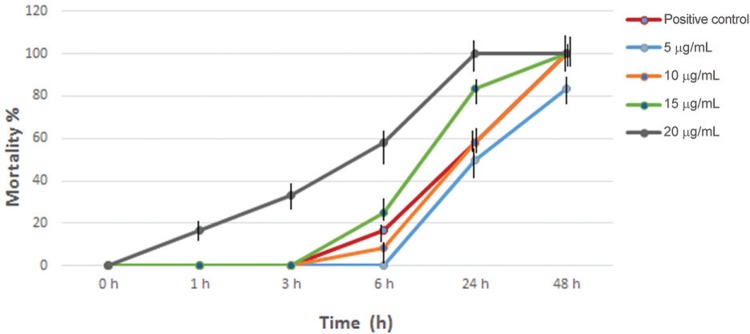
: mortality rate (%) of *Schistosoma mansoni* juvenile worms treated in vitro with different concentrations of primaquine for different incubation times*.* The mortality rate of both the negative control group and the group treated with 2 µg/mL of primaquine was 0%. Error bars represent the ± SD from three independent experiments.

**Fig. 2:**
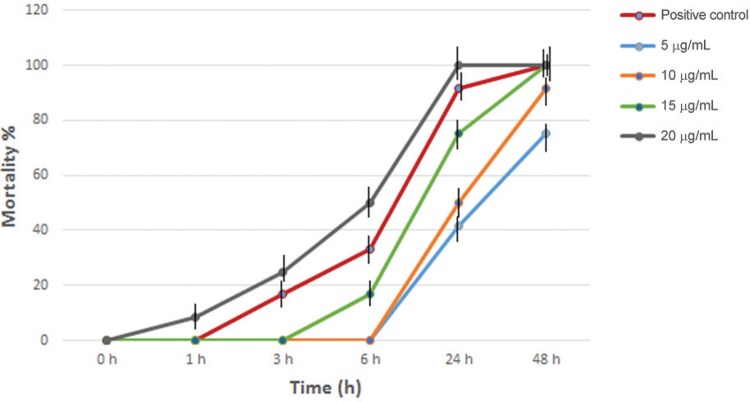
: mortality rate (%) of *Schistosoma mansoni* adult male worms treated in vitro with different concentrations of primaquine for different incubation times. The mortality rate of both the negative control group and the group treated with 2 µg/mL of primaquine was 0%. Error bars represent the ± SD from three independent experiments.

*Effect of primaquine on the mortality rate of S. mansoni juvenile and adult male worms in vitro* - The mortality rate of both juvenile (21 days old) and adult male worms (seven weeks old) at different incubation periods (1, 3, 6, 24, and 48 h) with different concentrations (2, 5, 10, 15, and 20 µg/mL) of primaquine were studied. As expected, all the worms (juvenile and adult male) incubated in pure medium (negative control) exhibited active movement and were alive throughout the experimental period of 48 h. The positive control group exposed to PZQ at a concentration of 5 µg/mL showed slow motility with a mortality rate of 58.3% in juvenile worms, whereas contraction and paralysis with a mortality rate of 91.7% was observed in adult male worms after 24 h of incubation. These effects progressed and became more intense as the time of incubation increased until the motility of all worms was reduced. The worms were considered dead after 48 h of exposure (Figs 1-2).

The mortality rate of both juvenile and adult male worms of *S. mansoni*, exposed to primaquine was also studied. At the concentrations 2, 5, and 10 µg/mL, the mortality rate in juvenile worms was 0%, 50%, and 58.3%, respectively after 24 h of incubation, whereas it was 0%, 83.3%, and 100%, respectively after 48 h of incubation. On the other hand, in adult male worms the mortality rate was 0%, 41.7%, and 50%, respectively after 24 h of incubation, whereas after 48 h of incubation it was 0%, 75%, and 91.7%, respectively. In addition, juvenile worms appeared slightly more sensitive to the schistosomicidal activity of primaquine than did the adult male worms at a concentration of 15 µg/mL. Meanwhile, the concentration of 20 µg/mL caused contraction, paralysis, followed by death of all worms after 24 h of exposure to the drug (data not shown). Parasite death was irreversible, as judged by the examination of worms following washing and overnight incubation in drug free medium. Thus, the mortality rate of the worms was directly proportional to both the concentration and the period of incubation (Figs 1-2).

**Fig. 3:**
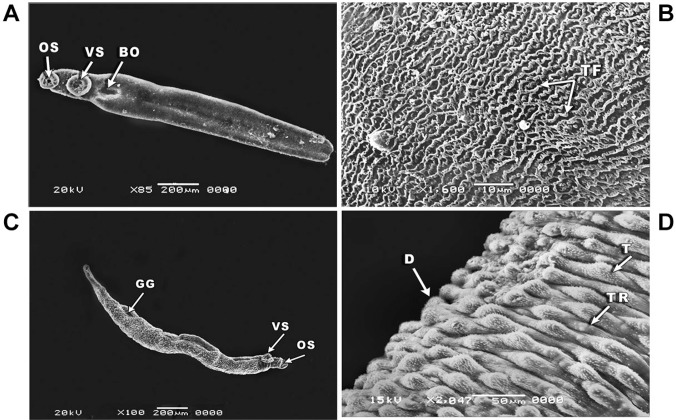
: scanning electron microscopy (SEM) of *Schistosoma mansoni* juvenile (A-B) and adult male worms (C-D) incubated in pure medium (negative control) for 24 h showing: (A) juvenile worm with an oral sucker (OS), ventral sucker (VS) and a bore (BO) marking the beginning of the gynaecophoric groove (×85); (B) the dorsal surface of the juvenile worm showing rows of tegumental folds (TF) (×1600); (C) adult male worm showing the oral sucker (OS), ventral sucker (VS), and gynaecophoric groove (GG) (×100); (D) adult male worm with well-developed tubercles (T) and tegumental ridges (TR) on the dorsal surface (D) (×2.047).

*Tegumental alternations in S. mansoni juvenile and adult male worms in response to primaquine as visualised by SEM* - Both juvenile (21 days old) and adult male *S. mansoni* worms (seven weeks old) incubated in pure medium (negative control) showed intact surface structure and topography (Fig. 3A-D). In the juvenile worm, the body consisted of oral and ventral suckers and a bore exhibited the beginning of the gynaecophoric groove (Fig. 3A), whereas the dorsal surface is covered with rows of tegumental folds (Fig. 3B). The adult male worm (negative control) showed oral and ventral suckers and the gynaecophoric groove on the ventral surface (Fig. 3C), with well-developed tubercles and tegumental ridges on the dorsal surface (Fig. 3D).

**Fig. 4:**
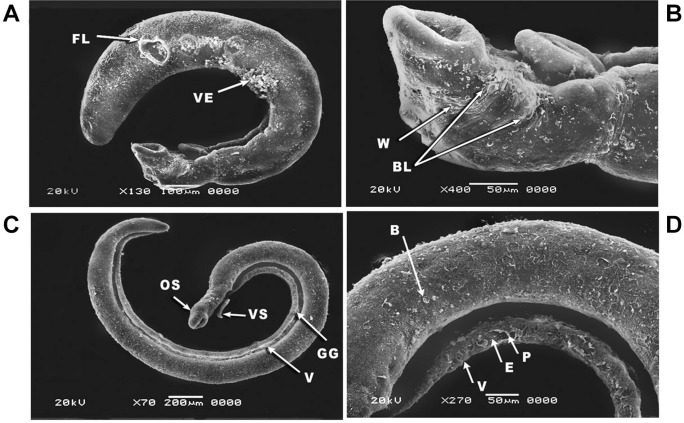
: scanning electron microscopy (SEM) of *Schistosoma mansoni* juvenile (A-B) and adult male worms (C-D) exposed to 5 µg/mL of Praziquantel (PZQ) (positive control) for 24 h showing: (A) juvenile worm contraction with vesicles (VE) and focal lesions (FL) in the middle part of body (×130); (B) magnification of the anterior region of a juvenile worm showing tegumental wrinkles (W) and blisters (BL) (×400); (C) adult male worm bending in the ventral (V) direction with oral sucker (OS), ventral sucker (VS), and gynecophric groove (GG) (×70); (D) ventral surface (V) of an adult male worm showing peeling (P) and erosion (E) of the tegument with blebs (B) (×270).

Tegument alterations were observed on *S. mansoni* juvenile and adult male worms after 24 h of incubation with a concentration of 5 µg/mL PZQ (positive control) (Fig. 4A-D). In the juvenile worm, the morphological alternations were demonstrated as contraction of the worm body with vesicles and focal lesions in the middle region of the worm (Fig. 4A). In addition, some wrinkles and blisters appeared in the anterior area of the juvenile worm (Fig. 4B). In the adult male worm, the entire body was bent ventrally (Fig. 4C) and the tegument exhibited extensive areas of peeling and erosion with blebs (Fig. 4D).

**Fig. 5:**
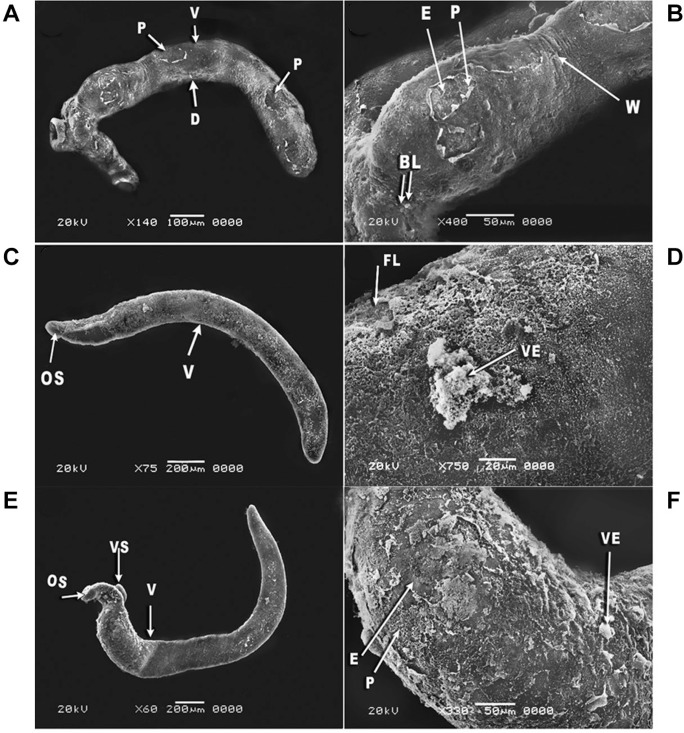
: scanning electron microscopy (SEM) of *Schistosoma mansoni* juvenile worms exposed to different concentrations of primaquine (A-F) for 24 h showing: (A) juvenile worm bending dorsally (D) with tegumental peeling (P) on the ventral surface (V) of the worm at concentration 10 µg/mL (×140); (B) magnification of the anterior region of a juvenile worm showing erosion (E), peeling (P) and blisters (BL) with tegumental wrinkles (W) at the concentration 10 µg/mL (×400); (C) juvenile worm with curving body in the ventral direction (V) showing oral sucker (OS) at the concentration 15 µg/mL (×75); (D) magnification of the anterior region of a juvenile worm showing focal lesions (FL) on the tegument with large vesicles (VE) at the concentration 15 µg/mL (×750); (E) juvenile worm with ventrally (V) bending body showing oral sucker (OS) and ventral sucker (VS) at the concentration 20 µg/mL (×60); (F) magnification of the anterior region of a juvenile worm showing erosion (E), peeling (P), and vesicles (VE) at the concentration 20 µg/mL (×330).

**Fig. 6:**
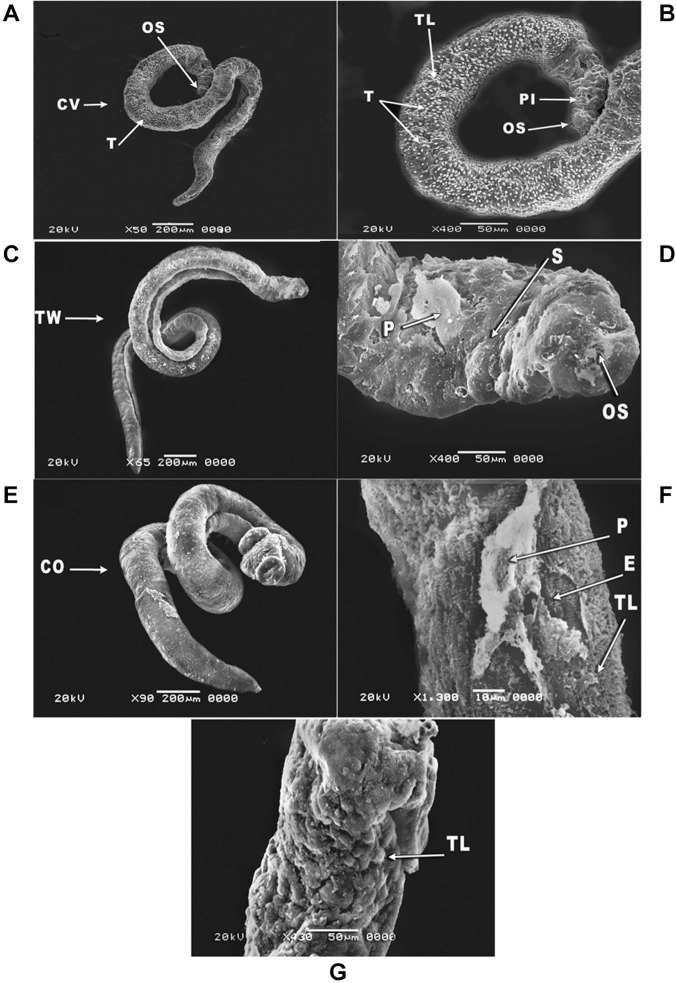
: scanning electron microscopy (SEM) of *Schistosoma mansoni* adult male worms exposed to different concentrations of primaquine (A-G) for 24 h showing: (A) convoluting (CV) of the adult male worm body with destruction of tubercles (T) at the concentration 10 µg/mL (×50); (B) magnification of the anterior region of an adult male worm showing pitted (PI) tegumental layer (TL), destruction of tubercles (T) and changes in the oral sucker (OS) at the concentration 10 µg/mL (×400); (C) twisting (TW) of the adult male worm at the middle region of the body at the concentration 15 µg/mL (×65); (D) magnification of the anterior region of adult male worm showing destruction of the oral sucker (OS) and sloughing (S) of the tegument with peeling (P) at the concentration 15 µg/mL (×400); (E) adult male worm showing coiling body (CO) at the concentration 20 µg/mL (×90); (F) magnification of the middle region of an adult male worm showing extensive peeling (P) and erosion (E) of the tegumental layer (TL) at the concentration 20 µg/mL (×1300); (G) adult male worm with destruction of the tegumental layer (TL) at the concentration 20 µg/mL (×430).

Twenty-four hours after exposure to primaquine at different concentrations, all the examined juvenile and adult male worms showed variable degrees of tegumental changes. Most of the juvenile worms incubated at the concentration of 10 µg/mL were characterised by dorsal bending of the worm body with tegumental peeling on the ventral surface (Fig. 5A). Moreover, the magnified anterior portion of the worm showed some erosion, peeling, blisters, and wrinkles (Fig. 5B). Additionally, at the concentration 15 µg/mL, the worms were curved ventrally and their anterior region showed focal lesions on the tegument with large vesicles (Fig. 5C-D). Furthermore, severe damages were observed at the concentration 20 µg/mL, where the worm was bent ventrally (Fig. 5E) with extensive erosion and peeling that extended along the entire dorsal worm surface as along with vesicles (Fig. 5F). In case of adult male worms incubated with primaquine at the concentration 10 µg/mL for 24 h, the worm body was convoluted with marked destruction of tubercles (Fig. 6A). Besides the pitting of the tegumental layer, changes in oral sucker were also observed (Fig. 6B). At the concentration 15 µg/mL, the worms showed twisting in the middle region along with destruction of the oral sucker and sloughing of the tegument (Fig. 6C-D). Additionally, coiling of the male worm body was observed at the concentration 20 µg/mL (Fig. 6E), with extensive peeling and erosion of the tegumental layer (Fig. 6F). Severe destruction of the tegumental layer was also observed (Fig. 6G). However, at low concentrations (2 and 5 µg/mL) no morphological alterations were observed. Thus, the specific morphological changes in juvenile and adult male worms were closely related to the drug concentration used.

**Fig. 7:**
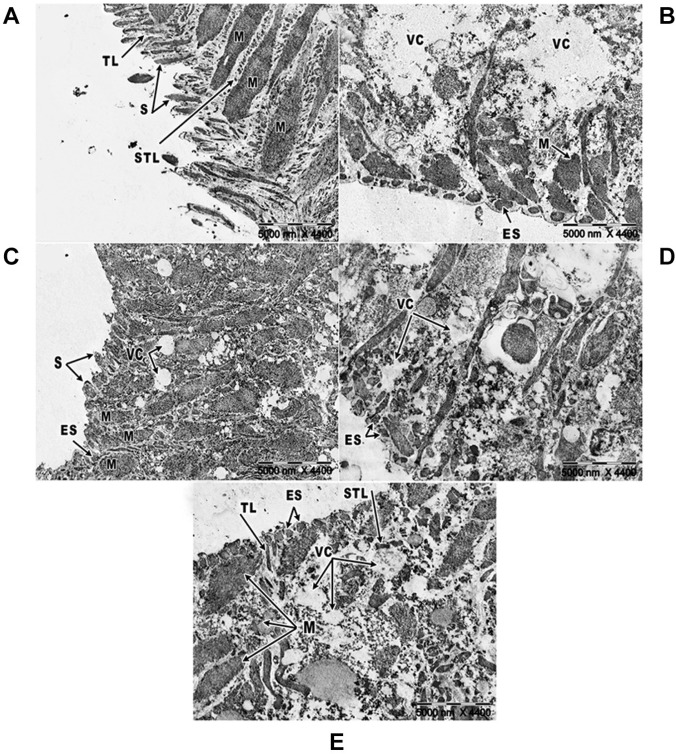
: ultrastructural alterations in adult male *Schistosoma mansoni* worms: (A) Adult male worm incubated for 24 h in pure medium (negative control) showing normal pointed spines (S), tegumental layer (TL), subtegumental layer (STL), and normal muscles (M) (×4400); (B) adult male worm maintained in a medium with Praziquantel (PZQ) (positive control) for 24 h showing embedded spines (ES) and shrinkage in muscles (M) with formation of vacuoles (VC) (×4400); (C) adult male worm exposed to primaquine for 24 h showing embedded spines (ES) and pointed spines (S) with small vacuoles (VC) between the normal muscles (M) at the concentration 10 µg/mL (×4400); (D) more embedded spines (ES) and a large vacuole (VC) in adult male worm at the concentration 15 µg/mL (×4400); (E) adult male worm with degeneration in both the tegumental (TL) and subtegumental layers (STL), embedded spines (ES), large vacuoles (VC), and shrinkage in the muscles (M) at the concentration 20 µg/mL (×4400).

*Effect of primaquine on the ultrastructural alternations of the S. mansoni adult male worm tegument using transmission electron microscopy (TEM)* - Since the ultrastructure of the tegument of juvenile *S. mansoni* worms was more or less similar to that of adult worms, the ultrastructural alterations in the tegument of *S*. *mansoni* adult male worms were studied. In pure medium (negative control), the adult male *S. mansoni* worm tegument contained numerous normal pointed spines with their triangular shaped covering the tegumental layer; normal muscles were also monitored in the subtegumental layer (Fig. 7A). The tegument of the *S. mansoni* adult male worm incubated with PZQ (positive control) for 24 h, revealed noticeable degeneration of the tegumental and subtegumental layers, embedded spines, and muscle shrinkage with widespread vacuolisation (Fig. 7B).

The tegumental surface of adult male worms incubated with different concentrations of primaquine showed damaged tegumental structure that varied from slight to severe. At 10 µg/mL of primaquine, some spines were embedded and some others were still pointed with disorganisation of the underlying muscles, accompanied by many small vacuoles (Fig. 7C). Moreover, more spines were completely embedded in the tegumental layer with more degeneration in the subtegumental layer and large vacuoles at the concentration 15 µg/mL (Fig. 7D). Increasing the concentration resulted in more alterations. At a high concentration (20 µg/mL), extensive damage of the tegumental and subtegumental layers with embedded spines was observed (Fig. 7E).

## DISCUSSION

Schistosomiasis is treated by the administration of PZQ, which exhibits good efficacy and low toxicity. Despite this, *S. mansoni* shows drug tolerance or resistance (Doenhoff et al. 2008). Therefore, it is important to develop new drugs for treating this disease, since PZQ is far from ideal (Lima et al. 2011). Recent discoveries have shown that the anti-malarial drug primaquine exhibited schistosomicidal activity against both juvenile and adult worms of *S. mansoni* in vitro (Holtfreter et al. 2011). The anti-schistosomal effects of primaquine are noteworthy as it is used for the treatment of malaria in schistosomiasis endemic areas (Holtfreter et al. 2011).

In this study, adult male worms were used instead of females, because females are not in direct contact with the host microenvironment. Earlier studies have shown that soft tissue alterations in male worms are more pronounced than those in female worms (Mostafa & Soliman 2002, Matos-Rocha et al. 2016).

Both scanning and transmission electron microscope play an important role in elucidating the detailed morphology and different alterations of the *S. mansoni* tegument allowing the interpretation of its functionality (Senft & Gibler 1977, El-Shabasy et al. 2015). These methods have been employed by several researchers (de Oliveira et al. 2012, El-Shabasy et al. 2015) and help to explain the mechanisms of action of anti-schistosomal drugs. The normal *S. mansoni* tegument is an essential interface between the parasite and the intravascular environment in the host (El-Shabasy et al. 2015).

Juvenile worms incubated with PZQ at 5 µg/mL for 24 h showed slow motion with a mortality rate of 58.3%. Moreover, the body was contracted with vesicles and focal lesions, as well as wrinkles and blisters. However, in the adult *S. mansoni* male worms, absence of movement as well as paralysis of the parasites was observed with a mortality rate of 91.7%. Moreover, the whole worm body was bent ventrally and the tegument exhibited extensive areas of peeling and erosion with blebs. These results were in accordance with those reported by Pica-Mattoccia and Cioli (2004), who demonstrated that PZQ caused both contractions and paralysis in adult worms of *S. mansoni* with little effect on juvenile worms at the concentrations 0.1 and 1 µg/mL. PZQ damaged the tegument, and facilitated eosinophilic granulocytes to damage the parasites. In addition, it led to contraction and paralysis accompanied by muscle spasms in the worm, caused by the influx of Ca^2+^ ions into the tegument (Shaw & Erasmus 1987).These finding are in parallel with those reported by Mehlhorn et al. (1981), who stated that the principle effect of PZQ finally led to the death of *S. mansoni* with disruption of the worm tegument. Additionally, the ultrastructural changes in adult male worms included degeneration of the tegument and subtegumental layers as well as shrinkage of the muscle with embedded spines. These findings were in accordance with those of El-Shabasy et al. (2015) who confirmed the same ultrastructural transformation of the tegument induced by PZQ.

This study revealed that the effects of primaquine were dose-dependent, and the most effective dose was 20 µg/mL in a short incubation period of 24 h. In addition, it was possible to observe that both juvenile and adult male worms exposed to the drug exhibited contraction, motility reduction, and paralysis resulting in death. The main changes induced by primaquine in the present study were at the concentrations 10, 15, and 20 µg/mL. In the juveniles, most of the changes included bending of the worm with tegumental erosion and peeling. Besides, blebs and wrinkles that extended to the entire dorsal surface of the worms as well as vesicles were also observed. The tegumental alternations in the adult male worms included convoluting of the worms, destruction of tubercles, and peeling and erosion of the tegumental layer. Furthermore, at low concentrations (2 and 5 µg/mL) no morphological alterations occurred. Mitsui and Aoki (2010) noticed that when *S. mansoni* adult worms were exposed to primaquine in vitro at the concentration 10 µg/mL, the survival time of the adult worm was reduced with the appearance of remarkable blebs on the body of the male worm accompanied by swelling and finally death. Similar results were observed by Holtfreter et al. (2011), who elucidated the damage caused by primaquine in vitro on adult worms of *S. mansoni*. This damage was manifested in loss of their attachment to the walls of the culture plates, possibly due to the damage of the muscles in both the oral and ventral suckers.

The present study showed that 24 h after incubation in primaquine at the concentrations 10, 15, and 20 µg/mL, severe ultrastructural damage occurred in adult male worms. The highest degree of damage was seen in both the tegumental and subtegumental layers. The tegument layer was embedded with spines, whereas the subtegumental layer showed shrinkage of muscles with vacuoles at the concentrations 10 and 15 µg/mL; thereafter, increasing the concentration to 20 µg/mL resulted in extensive damage including degeneration of both the tegumental and subtegumental layers.

There are no recent published studies concerning the ultrastructural changes induced by primaquine on *S. mansoni* adult male worms; the results of both scanning and transmission electron microscopy in the present work show that the schistosome tegument is the main target of primaquine. Several researchers have reported that the worm tegument is the key target for many anti-schistosomal drugs as mefloquine and Synriam™ (Manneck et al. 2010, Mossallam et al. 2015). In vitro assays cannot cover all aspects of drug anthelminthic activities, particularly pharmacological and immunological host interactions; however, they provide first evidence of anthelminthic effects and an insight into the mode of action, and they may lead to the development of new therapeutic drugs (Holtfreter et al. 2010). Considering the results obtained in this study, the schistosomicidal activity of primaquine indicates that this drug possesses moderate in vitro activity against juvenile and adult male *S. mansoni* worms, since it caused high schistosome mortality and pronounced morphological alterations. Therefore, this study opens up perspectives for future research on this drug in vivo to discover its mechanism of action.
